# Bipolar Disorder and Comorbid Use of Illicit Substances

**DOI:** 10.3390/medicina57111256

**Published:** 2021-11-17

**Authors:** Ulrich W. Preuss, Martin Schaefer, Christoph Born, Heinz Grunze

**Affiliations:** 1 Klinik für Psychiatrie, Psychotherapie und Psychosomatische Medizin, Klinikum Ludwigsburg, Posilipostrasse 4, 71640 Ludwigsburg, Germany; 2 Klinik für Psychiatrie, Psychotherapie und Psychosomatik, Martin-Luther-Universität, Halle-Wittenberg, Julius-Kühn-Str. 7, 06112 Halle/Saale, Germany; 3 Klinik für Psychiatrie, Psychotherapie, Psychosomatik und Suchtmedizin, Evang. Kliniken Essen-Mitte, Henricistr. 92, 45136 Essen, Germany; m.schaefer@kem-med.com; 4 Klinik für Psychiatrie und Psychotherapie (CCM), Charité Universitätsmedizin, 10117 Berlin, Germany; 5 Psychiatrie Schwäbisch Hall, 74523 Schwäbisch Hall, Germany; c.born@klinikum-weissenhof.de (C.B.); heinz.grunze@icloud.com (H.G.); 6 Campus Nuremberg-Nord, Paracelsus Medical University, 90419 Nuremberg, Germany

**Keywords:** bipolar disorders, substance use disorders, cocaine, cannabis, illicit drugs, mania, mood disorders

## Abstract

Substance use disorders (SUD) are highly prevalent in bipolar disorder (BD) and significantly affect clinical outcomes. Incidence and management of illicit drug use differ from alcohol use disorders, nicotine use of behavioral addictions. It is not yet clear why people with bipolar disorder are at higher risk of addictive disorders, but recent data suggest common neurobiological and genetic underpinnings and epigenetic alterations. In the absence of specific diagnostic instruments, the clinical interview is conducive for the diagnosis. Treating SUD in bipolar disorder requires a comprehensive and multidisciplinary approach. Most treatment trials focus on single drugs, such as cannabis alone or in combination with alcohol, cocaine, or amphetamines. Synopsis of data provides limited evidence that lithium and valproate are effective for the treatment of mood symptoms in cannabis users and may reduce substance use. Furthermore, the neuroprotective agent citicoline may reduce cocaine consumption in BD subjects. However, many of the available studies had an open-label design and were of modest to small sample size. The very few available psychotherapeutic trials indicate no significant differences in outcomes between BD with or without SUD. Although SUD is one of the most important comorbidities in BD with a significant influence on clinical outcome, there is still a lack both of basic research and clinical trials, allowing for evidence-based and specific best practices.

## 1. Introduction

Bipolar disorder (BD) is a common, severe and cyclic mental illness that presents with marked and unpredictable changes in mood and activity [1]. BD is a risk factor for addictions, both behavioral, such as gambling [2] and substance use disorders [3]. Besides a strong association between alcohol [4] and nicotine [5] dependence and BD [4], the abuse of other drugs, such as cocaine, amphetamines, opiates, cannabis, and prescription medications is also an important health concern in people with BD. When conceptualizing this article, the authors noticed that there is a fair number of reviews on alcohol use disorder in individuals with BD, including their own recent publication [1], whereas there is a relative paucity of up-to-date, comprehensive reviews on the subject of bipolarity and illicit drugs.

Substances reviewed in this article under the heading of “illicit drugs” include amphetamines, cannabis and cocaine as they are among the most frequently used substances and have been, at least to some degree and different from, e.g., opioids, studied in subjects with BD. However, what constitutes an illicit drug varies between countries, e.g., cannabis has been legalized in some countries but not in others. In this sense, the term “illicit drugs” for these substances applies to most countries, including the author’s home country Germany, but not to all.

Milder mood symptoms including depressive or euphoric mood swings may, in many instances, be the result of substance use. However, these subjects may also suffer from cyclothymic or bipolar II disorders (BD II). Moreover, extensive use of amphetamines or cocaine may also mimic manic symptoms or may be a risk factor for a switch into a manic episode in a primarily bipolar subject. Substance use and substance use disorders (SUD) may be associated with medication non-compliance, more mixed or dysphoric mania, and possibly an earlier onset of affective symptoms and more hospitalizations [6]. Thus, early recognition and treatment are of the utmost importance to improve long-term outcomes in people with BD. Improving mood symptoms by specific pharmacotherapy for BD may be the initial step to get a grip on drug use and use disorders, but in case of excessive drug consumption, acute detoxification treatment need to be first before specific BD treatments can be started. Mood stabilizers and/or atypical antipsychotics, as well as specific psychosocial interventions, may be useful for long-term stabilization in BD comorbid with SUD, including illicit drugs. This narrative review tries to summarize current knowledge about the incidence, influence and treatment of illicit drug abuse in people with BD.

## 2. Epidemiology of Illicit Substance Use in Bipolar Disorder

### 2.1. Results from General Population Surveys

The high association of BD with several substance use disorders (SUDs) has been consistently reported by epidemiological surveys and also clinical studies [7].

Recent epidemiological results on this co-morbidity have been reported from the National Epidemiologic Survey on Alcoholism and Related Conditions (NESARC) [8]. In a first wave (wave I), the survey interviewed around 43,093 people in 2001 and 2002. The rate of comorbid alcohol and substance-related disorders (AUD and SUD) in BD is, as expected, disproportionately high [9] and accomplish up to 50% for bipolar I disorder [10]. On the other hand, respondents with SUDs also have a higher lifetime rate of manic (3.7–13.4%) and hypomanic episodes (3.7–13.4%) [9] compared to the general population. In subjects with unipolar mania, also approx. 40% have been diagnosed with a comorbid SUD [11]. In comparison, rates for unipolar depression are 40.3% for AUD (21% for alcohol dependence) and 17.2% for other substance use disorders. More specifically, NESARC wave I found that females with mania had significantly higher odds ratios (OR) of any drug abuse, tranquilizer abuse, cocaine and opioid use disorders compared to males. Furthermore, compared to males, women with hypomania also had higher ORs of any SUD, including sedatives and opioid use disorders [11].

Regarding people with primary SUD, a high comorbidity rate of additional drug use disorders and antisocial personality disorder has been reported. However, there are also strong and important associations with mood and anxiety disorders. In their National Epidemiologic Survey on Alcohol and Related Conditions, Compton and colleagues showed that drug dependence over twelve months was significantly related to Major Depressive Disorders and BD-I, but interestingly not BD-II disorder. Lifetime treatment or help-seeking behavior was rare in SUD but significantly increased in case of psychiatric comorbidity [12].

### 2.2. Longitudinal Trends in Comorbid Illicit Substance Use Disorder and Bipolar Disorder

Subsequent analyses of the NESARC data (wave III, 2012–2013) reported rates and ORs of twelve month and lifetime prevalence of specific substance user disorders (SUD), including amphetamine, cannabis, club drug, cocaine, hallucinogens, heroin, non-heroin opioids, sedatives or tranquilizers, and solvent or inhalant use disorders [13]. Odds ratios for SUD in comorbid mental disorders were computed. Prevalence rates of 12-month and lifetime SUD for the total sample of *n* = 36,309 were 3.9% and 9.9%, respectively. Again, individuals with Bipolar I disorders had a significantly increased 12-months SUD OR (1.5; 95% CI, 1.06–2.05) and lifetime SUD OR (OR, 1.4; 95% CI, 1.14–1.74). In comparison, the bipolar II SUD associations were not statistically significant (12-month prevalence OR 1.3, 95% CI 10.64–2.69; lifetime prevalence OR 1.3, 95% CI 0.64–2.56). Among individuals with 12-month and lifetime SUD only a minority of 13.5% and 24.6% received treatment, respectively.

A recent meta-analysis reports on a prevalence of cannabis use of 24% (95% CI: 18–29; k = 35; *n* = 51,756) in people with BD. Cannabis use was significantly associated with being younger, male, and single; having fewer years of education and an earlier onset of affective symptoms [14].

These more recent epidemiological studies support previous results [7]. A large survey of major psychiatric disorders in five major metropolitan areas in the United States named the Epidemiological Catchment Area Study found that BD had the second-highest rate of SUDs compared to any other major psychiatric disorders, only topped by antisocial personality disorder [15]. Data of 463,003 patients were included in a large population-based cohort study of the prevalence of SUD among the Danish population [16]. The lifetime prevalence of SUD was almost one-third (32%) in patients with any BD. However, alcohol use disorder accounted for 25% of the prevalence rate of the SUDs.

A meta-analysis of epidemiological surveys on BD and SUD comorbidity between 2009 and 2014 included nine studies, of which two were repeated 10 years later in independent samples [3]. The sample size included *n* = 218,397 individuals. Strong statistical associations were detected between BD and illicit drug use (pooled OR 4.96; 95% CI 3.98–6.17). The association was higher for BD I individuals using illicit drugs compared to bipolar II respondents (ORs 7.48 vs. 3.30).

### 2.3. Comorbidity Rates of Substance Use Disorder and Bipolar Disorder in Clinical Settings

In a review and meta-analysis of clinical studies, 22 multi-site and 56 individual, mostly single-site studies reporting co-morbidity rates of SUD and BD in inpatients or outpatients were identified by systematic literature search [17]. The meta-analysis demonstrated that, next to alcohol (42%), the most frequent substance used in individuals with BD was cannabis (20%) followed by any drug use disorder, mostly cocaine and amphetamines (17%). Males had a higher lifetime SUD risk than females. BD with SUD had an earlier age at onset and a slightly higher rate of hospitalizations than BD without SUD. The highest rates of comorbid BD and SUD were reported in US samples, the lowest in Asian studies.

Of note, the influence of SUD on recovery from an episode of bipolar depression appears to be minimal according to a prospective US study. In a large outpatient treatment sample (STEP-BD: Systematic Treatment Enhancement Program for Bipolar Disorder), 2154 individuals with an index episode of bipolar depression completed the two years follow-up. Of these subjects, 1528 had no history of a SUD, while *n* = 468 (21.7%) had a past SUD and *n* = 158 (7.3%) had a current SUD. The median SUD age of onset was 19.0 ± 6.3 years (compared to comorbid alcohol use disorders of 18.6 ± 7.5 years). Survival analysis was applied to examine the time to recovery for each group and revealed that median recovery time in individuals with no SUD was 200 days, in subjects with past drug disorders 224 days and 184 days for those with current drug use disorders with no statistical significance across groups. However, those with current or past substance use disorder were more likely to experience a switch from depression directly into a manic, hypomanic, or mixed episode without a symptom-free interval [18], suggestive of greater mood instability in BD with SUD.

In summary, both epidemiological and clinical studies confirm the high co-incidence of drug use disorders in bipolar patients. On average, higher rates of SUD are reported in Bipolar I vs. Bipolar II patients. Some studies also report that cannabis is the most frequently consumed illicit substance in BD subjects while other illicit substance use disorders are less frequent, but still more common than in the general population.

## 3. Diagnosing Illicit Drug Use Disorder in Bipolar Subjects and Vice Versa

Whereas the Alcohol use disorder Identification Test (AUDIT) [19] appears a reliable instrument also in bipolar subjects [7,20], the diagnosis of comorbid SUD other than alcohol in individuals with bipolar disorder relies mainly on the clinical assessment and thus is subjective to the interviewer’s bias. The Drug Abuse Screening Test (DAST) [21] can support the diagnosis of SUD and appears reliable in a mixed sample of psychiatric outpatients [22], however, so far it has not been systematically examined in patients with BD.

The reverse is also true as it is often difficult to establish a firm bipolar diagnosis in people with illicit drug use, as both the short-term psychological and behavioral effects, e.g., euphoria, disinhibition, psychotic features, as well as the long-term consequences, such as depression, cognitive decline and personality changes mimic bipolar symptoms and are easily attributed to drug use without further exploration of bipolarity. Thus, it is important to delineate the temporal coincidence between behavioral changes and drug consumption, own history prior to the start of SUD, family history of mood disorders, etc. Instruments, such as the Hypomania Checklist (HCL-33 [23]) or the Mood Disorder Questionnaire (MDQ [24]) support the diagnostic procedure but may produce false positives in people with SUD.

## 4. Motivations and Consequences of Comorbid SUD in People with Bipolar Disorder

Overall, motives for consuming illicit drugs in individuals with BD do not differ from people with BD and primary SUD (SUD before the onset of BD). The most frequent reasons include improving mood, relieving tension, alleviating boredom, escape from reality, achieving/maintaining euphoria and increasing energy [25]. However, bipolar individuals might also seek relief by self-medication or even try to mimic hypomanic and manic states which they consider as the desirable mood state [26] (“addiction to mania”, meaning that the subjects experienced mania as a sensation that he/she wants to achieve and maintain as it gilds an otherwise pervasive lack of self-esteem.). The use of amphetamines or cocaine may induce or prolong manic periods with high levels of energy and excitement. During depressive episodes, stimulants are used as an attempt to alleviate depressive mood or low energy level. Sedatives are often consumed to numb sadness, anxiety symptoms, or hopelessness.

SUD in people with BD is associated with a multitude of negative consequences, influencing the course and prognosis of BD. In general, psychiatric comorbidity and especially drug abuse in BD is associated with a higher severity, expressed as more relapses, the worse overall course of the disease, reduced response to pharmacological treatments, such as lithium and also an increased risk for suicide attempts or suicide (see Table 1) [27,28,29]. In addition, cannabis use has been shown to strongly increase the risk of a first BD episode (OR 4.98) [30]. Substance abuse in early childhood can be an early symptom of BD, and also lead to misdiagnosis of a primary SUD. There are complex interactions between BD and SUD regarding time to first treatment. Lagerberg and colleagues showed that the risk of long treatment delays was increased in patients with BD and excessive substance use, defined as meeting criteria for a DSM-IV SUD. In primary BD, the risk for developing excessive substance use was increased in males, in patients with shorter education and but also in patients with longer treatment delays of BD [31]. In general, bipolar patients with a history of SUD were shown to be more likely to receive no or inadequate treatment for BD compared to bipolar patients without a history of addiction [32]. Furthermore, SUD is associated with more affective instability in bipolar subjects. In the Systematic Treatment Enhancement Program for Bipolar Disorder (STEP-BD) trial including 3750 bipolar subjects, a history of addiction increased the risk of switching into manic, hypomanic, or mixed phases after depression [18]. SUD also appears to increase the risk to commit violent crimes in individuals with BD. Fazel et al. [33] analyzed records of 3700 patients with BD compared to 37.000 controls over 30 years regarding the occurrence of violent crime in Sweden. 8.4% of individuals with bipolar committed violent crimes compared with 3.5% of general population controls. The risk was mostly confined to patients with substance use comorbidity (adjusted odds ratio (OR): 6.4) and was only minimal increased in patients without substance abuse comorbidity (adjusted OR: 1.3).

SUD is a major reason for an increased relative lifetime risk for chronic infectious diseases, such as HIV or chronic hepatitis C. Mathew and colleagues analyzed retrospectively collected data of 325,410 patients, seen between 1998 and 2004 within facilities and clinics of the Veterans Integrated Service Network, regarding HCV-Infection, comparing BD patients with and without SUD. Overall, all BD patients were at increased risk for HCV infection compared to controls. However, while patients with BD only had a relative risk (RR) of 1.36, the relative risk increased in patients with SUD (4.86) and even more in the group with BD and SUD (RR: 5.46) [34]. Table 1 summarizes the most detrimental effects of SUD in people with BD.

## 5. Treatment of Comorbid Bipolar Disorder and Substance Use Disorder

Compared to comorbid AUD and BD [4], much less is known about the optimized treatment of comorbid BD and illicit substance use. Given the wide variety and modes of action of illicit substances and drugs of dependence potential, treatment needs to be rather individual. Specific recommendations for pharmacotherapies with some level of evidence exist for BD with comorbid cannabis and cocaine use, and with a very low grade of evidence expert opinion, case series and open studies for heroin, amphetamine, methamphetamine, and polysubstance SUD comorbid with BD [35]. For psychotherapies and socio-therapies, recommendations are not substance-specific and focus more on the interplay between BD and addiction in general.

### 5.1. Pharmacotherapies of Comorbid BD and Illicit Substance Use

The 2012 Canadian Network for Mood and Anxiety Treatments (CANMAT) recommends adding valproate to lithium in BD patients with cannabis or cocaine use disorder [35], based on open and retrospective studies [36,37,38,39]. In 2019, Coles and coworkers published a systematic review including open and controlled studies in BD comorbid with SUD, ranging from tobacco to opioids, with the majority looking into AUD [40]. Most studies are open, non-randomized and have small to moderate sample sizes. However, there are also a few randomized, controlled studies that included comorbid BD subjects with cannabis, cocaine, amphetamine or opioids use, summarized in Table 2. Firm conclusions or recommendations, however, are almost impossible as the majority of trials included people with diverse SUD without differentiating results according to the substance of abuse. There is only one RCT conducted in methamphetamine-only users, two in cocaine-only users and none in cannabis-only users. If at all, the only conclusions with some degree of certainty in BD co-morbid with illicit substance use disorder are that (1) lithium and valproate are effective for mood symptoms and may reduce substance use (probably as an indirect effect due to improved mood stability), and (2) that the neuroprotective agent citicoline reduces cocaine consumption.

A small (*n* = 22) placebo-controlled, crossover, multimodal-MRI pilot study, linking neurobiology and clinical outcomes, reported on a potential benefit of gabapentin (1200 mg/d) in BD with comorbid cannabis use disorder [43]. Subjects taking gabapentin and having elevations of dorsal anterior cingulate cortex GABA levels in the MRI experienced lower manic/mixed and depressive symptoms. Elevations of right basal ganglia glutamate levels and posterior midcingulate cortex activation to cannabis cues were also associated with lower cannabis use in participants randomized to gabapentin [43]. However, large confirmative trials supporting the use of gabapentin in BD with comorbid cannabis use disorder are still missing, and the effectiveness of gabapentin in BD without SUD and in cannabis use disorder is equivocal or vague [50,51,52].

More recently and based on the proposed neurobiological aberration in SUD and BD, D2/D3 receptor partial agonists attract attention as a possible medication of interest. A recent case report on cariprazine an effective medication in BD [53]—in methamphetamine use disorder [54] argues for further evaluation in RCTs.

Of note, and contrasting its observed detrimental effects in BD subjects [55,56], some investigators also suggest a potential therapeutic role for cannabis derivative in BD [57]. The endocannabinoid system is known to exert neuro-modulatory effects on other neurotransmitter systems critical in controlling emotions [58]. Selective activation of the cannabinoid receptor 2 (CB2) and antagonism of cannabinoid receptor 1 (CB1) may alleviate the symptoms of BD and, according to Arjmand and colleagues [59], should be rigorously explored. This is in line with genetic findings suggesting that carriers of a specific variant of CB1 are also at higher risk of developing BD [60].

### 5.2. Psychosocial Therapies of Comorbid BD and Illicit Substance Use Disorders

Over the last decades, specific psycho and socio-therapeutic interventions have been developed and tested for BD comorbid with SUD; however, they target almost exclusively BD with comorbid AUD [4]. Different from the traditional paradigm to achieve complete abstinence first before focusing on mood, it appears nowadays to be common sense that group and individual integrated psychotherapies which address both disorders are more effective than interventions focusing on either disorder alone [7,20]. To define the focus of psychotherapy in an individual bipolar client with comorbid SUD it is important to understand how clients perceive the relationship between BD and SUD. The study by Healey and colleagues [61] relates the use of substances to either decrease or intensify mood symptoms, cope with living with BD, feel normal, or manage stress. Other factors reported by bipolar patients are related to self-medication, feelings of increased confidence, rejection of prescribed medication, easy access and living in a culture of substance use [62]. The findings from these qualitative studies are in line with those from a cross-sectional study that found that people with BD use substances to improve mood, relieve tension, alleviate boredom, achieve or maintain euphoria, and increase energy [25]. When subjects with comorbidity were asked what they consider as important for achieving recovery, support, individualized treatment, hope, a new sense of identity and having meaningful daily activities were considered as essential [63], and may set agenda and goals for psychotherapeutic interventions.

A recent systematic review by Crowe and colleagues [64] identified only five studies of psychotherapy for BD and two studies of integrated psychotherapy for comorbid BD and SUD that were methodologically acceptable to be included in their review. Inclusion criteria were randomized controlled trials of psychotherapy as an adjunct to medication, individual and group interventions, manualized interventions, English language of the papers which were published until November 2019. Of these studies, none focused on BD with comorbid illicit drug use but on SUD in general, in most instances AUD. There was a considerable variation in type and duration of approaches: Individual and group therapy, type of intervention (Interpersonal and social rhythm therapy (IPSRT), Systematic Treatment Enhancement Program for Bipolar Disorder (STEP-BD) intensive psychotherapy (cognitive-behavioral therapy (CBT), IPSRT, family focused therapy or collaborative care), CBT and integrated group therapy. The duration ranged from 12 weeks to 27 months [64], and all studies investigated only mood-related outcomes, but not changes in SUD measures. The good news is that when trying to summarize the main findings of the studies, it appears that the intuitive hypothesis that SUD delays recovery and promotes recurrences of mood episodes cannot be positively proven; most studies indicate no significant differences between BD with or without SUD. Again, these results are mainly derived from BD patients with AUD with only a minority using other substances.

Evidence, but not specific for BD, that psycho-social therapies might also ameliorate substance use came from a randomized clinical trial of a six-month, twice-weekly program, named “Behavioral treatment for drug abuse in people with severe and persistent mental illness” (BTSAS) program [65]. The BTSAS program is a social learning intervention that includes motivational interviewing, a urinalysis contingency, and social skills training. One hundred and twenty-nine affectively stabilized outpatients meeting DSM IV criteria for drug dependence (cocaine, heroin, or cannabis) and serious mental illness (39.5% with schizophrenia or schizoaffective disorder; 55.8% major affective disorders including BDs) were included and received either BTSAS or a supportive group discussion treatment (STAR as a control condition). Primary outcome measures were abstinence verified by twice-weekly urine analysis and time until dropping out of treatment (dropout defined as missing eight consecutive sessions). The BTSAS program was significantly more effective than STAR in the percentage of clean urine test results, survival in treatment, and attendance at sessions. Post hoc, exploratory analyses on a number of ancillary clinical outcomes demonstrated a significant decline in the number of hospitalizations, more money available for living expenses, and an increase in general life satisfaction.

## 6. Conclusions

Drug and alcohol abuse in subjects with severe and chronic mental illness, such as BD, is one of the major challenges the public mental health system has to deal with. These people pose major problems not only for themselves, but also their social environment, including family, friends, health care professionals, and the mental health system. The lifetime prevalence of substance use disorders has been estimated up to 56% for subjects with BD. AUD is most prevalent, but figures of approximately 25% for substance use other than alcohol and tobacco, mainly cannabis and cocaine, are also substantial. SUD tends to begin early in the course of BD, complicates and delays the correct diagnosis and has a profound impact on almost every domain of functioning and needs. SUD in BD predicts a more severe course of illness, more frequent hospitalizations, more frequent relapses, and a poorer course of illness in comparison to BD subjects without SUD. BD with SUD is associated with higher rates of violence and suicide. Finally, higher rates and costs of service utilization, as well as poorer treatment adherence resulting in inferior treatment outcomes call for the need for intensive and comprehensive treatment programs.

The treatment of patients with dual disorders requires an inclusive and multidisciplinary approach, integrating both psychiatric and substance abuse treatment. Ideally, and similar to BD with comorbid AUD, treatment of BD and illicit drug use disorder should be in parallel, not successive, in one place and coordinated by a named, accessible and responsible care coordinator [66]. Treatment should be understood as a process being in flux in which the motivation to reduce substance use might change and that needs an integrated treatment agenda and setting addressing both disorders. Finally, a harm-reduction model appears more appropriate than an abstinence model, especially during the early stages of treatment when the patient has an uncertain motivation for change [65]. The pharmacological treatment should focus on stabilization of mood after detoxification rather than focusing on substance abstinence. Psychotherapy, however, should include both; therapies for BD should be combined with behavioral treatments for drug abuse and abstinence tests with urine analysis. Although illicit drug use disorders are one of the most frequent comorbidities in BD and significantly influences clinical outcome, there are still only a few studies focusing on neurobiological commonalities, or RCTs that have investigated the effectiveness of psychotherapeutic or pharmacological treatments for BD with illicit drug use comorbidity. The fact that bipolar subjects with a history of SUD are usually excluded from clinical trials significantly limits the generalizability of RCT findings to the ‘real life’ patient with BD. Specific research is clearly needed to develop better treatment strategies in BD with illicit drug use.

## 7. Limitations

This narrative review summarizes current knowledge regarding illicit drug use in individuals with BD. The review focuses on illicit drug use, and therefore, does not include data about AUD, cigarette smoking, or the field of behavioral addictions, such as gambling disorder, which is also prevalent in subjects with BD. The review is mainly based on expert opinion and is not a systematic review including a systematic search of the literature. Thus, there is a chance that relevant contributions and articles have been missed.

## Figures and Tables

**Table 1:** Summary of the detrimental impact of comorbid drug abuse on BD.

diagnostic problems	worse compliance/adherence
earlier onset of illness	worse response/outcome
no treatment or treatment delay	more frequent relapses with manic and depressive episodes
increased psychosocial problems (job, debts...)	accelerated progression of BD
poorer social support	more negative and cognitive symptoms
increased severity of disease	increased risk for suicide attempts or suicide
somatic comorbidities (Hepatitis C, other liver diseases, Acquired Immune Deficiency Syndrome (AIDS), …)	increased risk of switching into manic, hypomanic, or mixed phases after depression
more rapid cycling	reduced response to lithium and other pharmacological treatments

**Table 2:** Randomized, controlled pharmacotherapy studies in BD comorbid with SUD (other than AUD or tobacco use disorder).

Substance	Study	Substances	Intervention	Bipolar Diagnosis and N	Design	Outcome/Limitations
**Amphetamines**	Nejtek et al., 2008 [41]	Methamphetamine or cocaine	Quetiapine or Risperidone	BD I or II. *N* = 80	20 weeks DB, add on to anticonvulsants or antidepressants. No PLC control.	See heading “Cocaine”. No separate results were reported for methamphetamine.
	Brown et al., 2012 [42]	Methamphetamine	Citicoline	BD I, II and BD-NOS, MDD currently depressed. *N* = 48	12 weeks DB, PLC controlled add-on to TAU	Depressive symptoms (IDS-score) ↓with citicoline. No significant differences in methamphetamine use between groups. Small and heterogenous sample.
**Cannabis**	Prisciandaro et al., 2021 [43]	Cannabis	Gabapentin	BDI or II and current (within the past 3 months) moderate-to-severe cannabis use disorder *N* = 22	GBP or PLC, 2-week RCT with cross-over after one week, add on to TAU. MRI study.	Primary outcome: 1H-MRS glutamate and GABA levels. GBP↑ dACC glutamate levels in participants with lower levels of substance use and mood symptoms. GBP**↑** rBG glutamate levels and pMCC activation to cannabis cues in cigarette-smoking participants. ↑rBG glutamate and dACC GABA levels in participants while on GBP were associated with ↓cannabis use and mood symptoms in those with more severe SUD and mood symptoms
	Gao et al., 2017 [44]	Cannabis, Alcohol or both	Quetiapine XR	BD I and II, currently depressed + comorbid anxiety. *N* = 90, but only 34 with cannabis use disorder	8 weeks DB, PLC Controlled add-on to MS	No significant difference between PLC and quetiapine XR in mood or substance use outcomes.
	Kemp et al., 2009 [45]	Alcohol, cannabis, cocaine	Lithium vs. lithium + valproate	Rapid cycling BD I or II Phase 1: *N* = 149; Phase 2: *N* = 31	6 months open label stabilization followed by 6 months DB RCT.	Phase 1 (open stabilization): Of the 15 subjects with cannabis use disorders, 53% no longer met the criteria for active cannabis abuse or had entered into early full remission. Of the 9 subjects with cocaine use disorders, 78% no longer met the criteria for active cocaine abuse or had entered into early full remission. Compared to baseline, a significant ↓ in mood symptoms was observed for both interventions. Phase 2 (DB RCT): No differences in mood outcomes between interventions.
	Geller et al., 1998 [46]	Alcohol, Cannabis, Inhalants	Lithium	Adolescents with BD I or II, single manic episode, or MDD with at least one predictor of future BD. *N* = 25, 2 on cannabis only and 14 on cannabis + alcohol	6 weeks DB, PLC controlled RCT add on to TAU	The lithium group showed significant↓ across mood and substance use outcome measures, compared to placebo. Small sample size, no separate outcome data reported for cannabis.
**Cocaine**	Nejtek et al., 2008 [41]	Cocaine or methamphetamine	Quetiapine or Risperidone	BD I or II. *N* = 80	20 weeks DB, add on to anticonvulsants or antidepressants. No PLC control	Both study medications were associated with a significant ↓ in manic, depressive, or mixed mood states compared to baseline scores. Both medications were also associated with ↓ drug cravings and use. Limited evidence in the absence of a PLC control. No separate results were reported for cocaine.
	Kemp et al., 2009 [45]	Alcohol, cannabis, cocaine	Lithium vs. lithium + valproate	Rapid cycling Bipolar I or II disorder. Phase 1: *N* = 149; Phase 2: *N* = 31	6 months open label stabilization followed by 6 months DB RCT.	See heading “Cannabis”. Phase 1 (open stabilization): Of 9 subjects with cocaine use disorders, 78% no longer met criteria for active cocaine abuse or had entered into early full remission.
	Brown et al., 2012 [47]	Cocaine	Lamotrigine	BD I, II, BD-NOS and cyclothymia, depressed or mixed, *N* = 120	10 weeks DB, PLC controlled add-on to TAU	No differences in mood outcomes and craving between lamotrigine and placebo. The lamotrigine group showed a greater ↓ in amount spent on cocaine.
	Brown et al., 2007 [48]	Cocaine and at least on other SUD	Citicoline	BD I or II (history of at least one (hypo)manic episode. *N* = 44	12 weeks DB, PLC controlled add-on to TAU	There were no significant changes in psychiatric symptoms between groups. A significant ↓ in the number of cocaine-positive urine screens was observed.
	Brown et al., 2015 [49]	Cocaine	Citicoline	BD I (depressed or mixed mood state) *N* = 130	12 weeks DB, PLC controlled add-on to TAU	Citicoline ↓ active cocaine use and the likelihood of relapse. There was no significant difference in craving symptoms between groups. No significant changes in mood symptoms.

BD I: Bipolar I disorder; BD II: Bipolar II disorder; BD NOS: Bipolar disorder not otherwise specified; dACC: dorsal anterior cingulate cortex; DB: Double-blind; GBP: Gabapentin; IDS: Inventory of depressive symptoms; MDD: Major depressive disorder; PLC: Placebo; pMCC: posterior midcingulate cortex; rBG: right basal ganglia; RCT: Randomized controlled trial; TAU Treatment as usual; ↓ indicates decrease; ↑ indicates an increase.

## Data Availability

All data mentioned in this review have been previously published and are in the public domain.
